# Parental use of the term "Hot Qi" to describe symptoms in their children in Hong Kong: a cross sectional survey "Hot Qi" in children

**DOI:** 10.1186/1746-4269-2-2

**Published:** 2006-01-05

**Authors:** Flora Y Kong, Daniel K Ng, Chung-hong Chan, Wan-lan Yu, Danny Chan, Ka-li Kwok, Pok-yu Chow

**Affiliations:** 1 Department of Paediatrics, Kwong Wah Hospital, 20 Waterloo Road, Kowloon, Hong Kong; 2 Department of Physiotherapy, Kwong Wah Hospital, 20 Waterloo Road, Kowloon, Hong Kong

## Abstract

**Background:**

The Chinese term "Hot Qi" is often used by parents to describe symptoms in their children. The current study was carried out to estimate the prevalence of using the Chinese term "Hot Qi" to describe symptoms in children by their parents and the symptomatology of "Hot Qi".

**Method:**

A cross sectional survey by face-to-face interview with a semi-structured questionnaire was carried out in a public hospital and a private clinic in Hong Kong. The parental use of the term "Hot Qi", the symptoms of "Hot Qi" and the remedies used for "Hot Qi" were asked.

**Results:**

1060 pairs of children and parents were interviewed. 903 (85.1%) of parents claimed that they had employed the term "Hot Qi" to describe their children's symptoms. Age of children and place of birth of parents were the predictors of parents using the term "Hot Qi". Eye discharge (37.2%), sore throat (33.9%), halitosis(32.8%), constipation(31.0%), and irritable (21.2%) were the top five symptoms of "Hot Qi" in children. The top five remedies for "Hot Qi" were the increased consumption of water (86.8%), fruit (72.5%), soup (70.5%), and the use of herbal beverages "five-flower- tea" (a combination of several flowers such as *Chrysanthemum morifolii*, *Lonicera japonica*, *Bombax malabaricum*, *Sophora japonica*, and *Plumeria rubra*) (57.6%) or selfheal fruit spike (*Prunella vulgaris*) (42.4%).

**Conclusion:**

"Hot Qi" is often used by Chinese parents to describe symptoms in their children in Hong Kong. Place of birth of parents and age of the children are main factors for parents to apply the term "Hot Qi" to describe symptoms of their children. The common symptoms of "Hot Qi" suggest infections or allergy.

## Background

As an ex-British colony in China, Hong Kong sees a thriving practice in both Western and Traditional Chinese medicine (TCM). Chinese often adopt the jargons of TCM to describe the symptoms. It is the authors' observation that the Chinese term "Hot Qi" is often used by parents to describe symptoms in their children. The term "Hot" is used by TCM practitioners to describe a phenomenon but not a diagnosis per se. [1,2] A better understanding of the meaning of "Hot Qi" would help western medical practitioners working with Chinese patients. So far, there has been no published data on this phenomenon. Parental use of the term "Hot Qi" to describe symptoms is not limited to those residing in Asia, but also for Asian immigrants in around the world. Understanding patient's symptom by health professionals helps to avert problems and misunderstanding, improve satisfaction for all parties and lead to better outcomes. [3]

The current study was undertaken to find out the frequency of using the term "Hot Qi" in parents and the symptomatology of the term "Hot Qi". This included a questionnaire on the symptoms and treatments for "Hot Qi". We hypothesized that the term "Hot Qi" was used among the majority of Hong Kong Chinese parents to describe symptoms in their children and the usage was governed by the cultural background of parents and status of children, e.g. age and gender.

## Methods

### Subjects

All parents of patients who were in-patients or attending outpatient clinics of our department, a private paediatric clinic, as well as the nursing and secretarial staff of this hospital were recruited for the study. Parents were approached individually by one of the authors (FYK) and the purpose of the survey was explained. A semi-structured interview based on the questionnaire (see Additional file 1) was conducted in Cantonese. Exclusion criteria include non-Chinese speakers and absence of parents.

The current study did not involve any medical intervention nor invasive intervention to the subjects and no ethical approval was deemed necessary under Hong Kong ethical framework.

### "Hot Qi" questionnaire

Parents were asked to express their view on the term "Hot Qi" as used on their children, one child per parent, based on a questionnaire (appendix 1). This questionnaire was developed by DKN and DC (a registered TCM practitioner). "Hot Qi" was investigated with the question: "Have you ever used the term "Hot Qi" to describe your child?" Parents who answered "yes" were asked to volunteer symptoms of "Hot Qi" that their children displayed and the remedies used.

### Statistics

All analysis was done with statistical software (Statistical Package for the Social Science, release 11.0.4 for Macintosh; SPSS; Chicago, IL). All continuous data were presented as mean and standard deviation. Age of children was compared between those who used the term "Hot Qi" and those who did not by Mann-Whitney U test. The categorical variables, included age group of parents, gender of parents, education level of parents, place of birth of parents, household income, gender of children were compared by Chi-squared test. Variables with significant difference between the two groups of parents were entered into a forward logistic regression model to predict the use of "Hot Qi" in parents. The demographical predictors of "Hot Qi" with adjusted odds ratio significantly larger than one were reported. Top five symptoms of "Hot Qi" and Top five remedies of "Hot Qi" were reported.

In those who reported use of the term "Hot Qi", we compared the mean age of children between those used and those who did not use a specific remedy by unpaired t-test. "Consumption of herbal products" was defined as any positive answer for "Five-flower-tea" (a combination of several flowers such as *Chrysanthemum morifolii*, *Lonicera japonica*, *Bombax malabaricum*, *Sophora japonica*, and *Plumeria rubra*), "selfheal fruit spike" (*Prunella vulgaris*), appetite stimulant (A brew from malt, juncus, and bomboo leaves), Abrus herb (*Abrus precatorius*), Turtle Jelly, Mulberry leaf & Chrysanthemum flower tea, Bo Ying Compound (a generic TCM product with the ingredients: Moschus, *Calculus Bovis*, *Borneolum syntheticum*, Margarita, *Lapis micae aureus*, Alumen, Succinum, *Herba ephedrae*, *Arisaema cum bile*, *Concretio silicea bambusae*, *Rhizoma paridis*, *Radix saposhnikoviae*, *Rhizoma pinelliae*, *Bulbus Fritillariae cirrhosae*, Scorpion, *Rhizoma coptidis*, *Bombyx batryticatus*, *Ramulus uncariae cum Uncis*, *Radix curcumae*, *Herba menthae*, *Rhizoma gastrodiae*, *Periostracum cicadae*), 24 taste herb tea, instant chrysanthemum tea and "Yin Chiao Chieh Tu Pien" (a generic TCM product with the ingredients: honeysuckle flower, forsythia fruit, platycodon root, peppermint, bamboo leaf, licorice root, schizonepeta, burdock root and black soybean). All significance tests were two sided, and a p < 0.05 was considered statistically significant.

## Results

1106 pairs of children and parents were approached for the survey and 46 parents refused to be interviewed. (Total number of parent interviewed: 1060, response rate = 96.8%) Nine hundred and three parents (85.2%) claimed that they had employed the term "Hot Qi" to describe their children's symptoms. Characteristics of the two groups of parents that used or did not use the term "Hot Qi' by parents were listed in Table 1.

**Table 1:** Characteristics of two groups of parents and their children that use or did not use the term "Hot Qi"

Characteristics	Use the term "Hot Qi"	Not use the term "Hot Qi"	Total	P *
Age of Parents				<0.001
<30	113 (71.1%)	46 (28.9%)	159	
30 – 40	429 (85.3%)	74 (14.7%)	503	
40 – 50	274 (89.0%)	34 (11.0%)	308	
50 +	87 (96.7%)	3 (3.3%)	90	

Gender of Parents				0.646
Male	150 (83.8%)	29 (16.2%)	179	
Female	753 (85.5%)	128 (14.5%)	881	

Education Level of Parents				0.186
Primary	138 (89.6%)	16 (10.4%)	154	
Secondary	620 (84.0%)	118 (16.0%)	738	
College/University	145 (86.3%)	23 (13.7%)	168	

Place of Birth of Parents				0.013
Hong Kong	604 (87.5%)	86 (12.5%)	690	
Mainland	280 (80.9%)	66 (19.1%)	346	
Others	19 (79.2%)	5 (20.8%)	24	

Monthly Household Income				0.158
(USD)	26 (83.9%)	5 (16.1%)	31	
On social security	137 (80.1%)	34 (19.9%)	171	
<1281	287 (82.7%)	60 (17.3%)	347	
1282 – 2564	223 (88.1%)	30 (11.9%)	253	
2565 – 3846	100 (87.7%)	14 (12.3%)	114	
3847 – 5128	74 (90.2%)	8 (8.8%)	82	
5129 – 7692	56 (90.3%)	6 (9.7%)	62	
> 7693				

Median age of Children (IQR)	7.0 (3.0 to 12.0)	1.0 (0.42 to 6.25)	6 (2 to 12)	<0.001

Gender of Children				0.299
Male	497 (84.1%)	94 (15.9%)	591	
Female	406 (88.1%)	55 (11.9%)	461	

All data are presented as number (percentage) unless otherwise indicated

* Compared between "Hot Qi" and "Without Hot Qi"group

**Figure 1:**
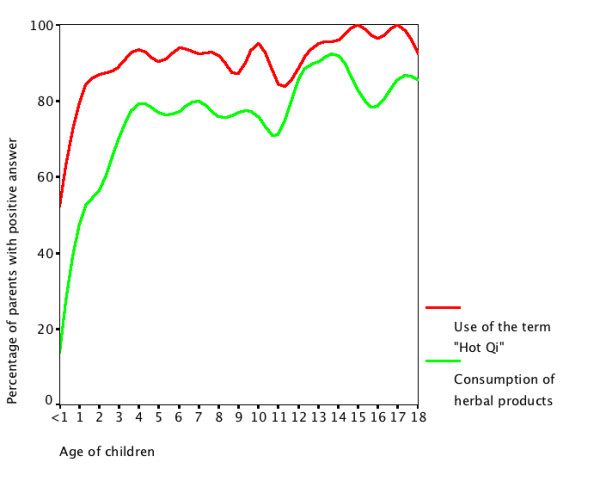
Age of children and the percentage of parents with positive answer of "Hot Qi" or "Consumption of herbal products".

There was no significant difference in gender, education level, and monthly household income distribution between the group of parents who employed the term "Hot Qi" and those who did not. Among children whose parents employed the term "Hot Qi" to describe them, there were 469 girls (44.2%) and mean age were 8.5 years (SD = 6.6). The children whose parents have used the term "Hot Qi" were significantly older. The frequency of the use of the term "Hot Qi" increased with older children. (Figure 1)

In univariate analysis (Table 1), age of parents, place of birth of parents and age of children were significantly associated with the frequency of using the term "Hot Qi". They were further analyzed by logistic regression analysis (Table 2) and only place of birth and age of children were significantly associated with the frequency of using the term "Hot Qi". Parents who were born in Hong Kong were 1.53 times more likely to use the term 'Hot Qi' to describe their children than parents born in mainland China and older children were more likely to be labeled as having "Hot Qi". Children older than or equal to 2-year were 6.79 times (95% CI = 4.73 to 9.76) more likely to report "Hot Qi" than children younger than 2-year.

**Table 2:** Significant risk factor of "Hot Qi" by using logistic regression analysis

Variables	B (SE)	P	Adjusted Odds ratio (95% CI)
Place of birth of parents (China) – Reference	0	0.031	1
Place of birth of parents (Hong Kong)	0.423 (0.184)	0.022	1.53 (1.06 to 2.19)
Place of birth of parents (Other)	-0.438 (0.541)	0.418	0.65 (0.22 to 1.86)
Age of children	0.132 (0.019)	<0.001	1.14 (1.09 to 1.19)

* Total percentage of variance explained by this model = 11.5 %

### Symptoms of "Hot Qi" and remedies used

**Figure 2:**
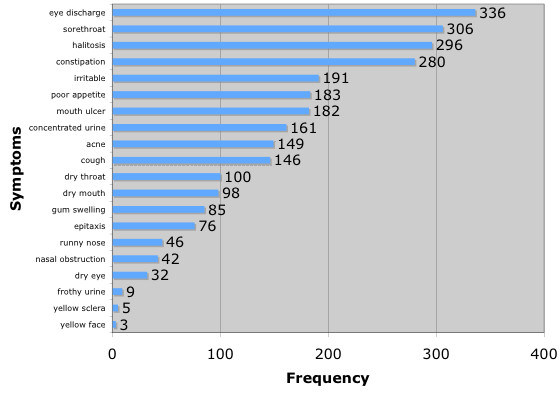
Frequency of symptoms reported by parents.

Symptoms of "Hot Qi" were summarized in Fig. 2. Among the symptoms of "Hot Qi" : eye discharge (37.2%), sore throat (33.9%), halitosis (32.8%), constipation (31.0%), and irritability (21.2%) were most frequently mentioned by parents. (Figure 2)

We studied the reasons for irritability in children (Table 3) because irritability is very likely to be secondary to other symptoms in children. Dry eye, nasal obstruction, poor appetite, dry mouth and dry throat were significantly associated with irritability in children reported to have Hot Qi.

**Table 3:** Risk factors for irritability in children with Hot Qi, univariate and multivariate analysis.

Variables	Crude Odds ratio (95% CI)	Adjusted odds ratio (95% CI)
Dry eye	6.82 (3.27 to 14.23) *	3.73 (1.45 to 9.59) *
Eye discharge	2.28 (1.65 to 3.15) *	1.34 (0.92 to 1.97)
Epitaxis	1.70 (1.01 to 2.86) *	0.83 (0.43 to 1.60)
Nasal obstruction	4.50 (2.40 to 8.44) *	2.65 (1.19 to 5.88) *
Runny nose	1.33 (0.68 to 2.64)	-
Gum Swelling	2.97 (1.86 to 4.73) *	1.67 (0.91 to 3.01)
Poor appetite	6.56 (4.58 to 9.40) *	5.39 (3.57 to 8.15) *
Yellow face	NA **	-
Yellow sclera	5.67 (0.94 to 34.15)	-
Concentrated urine	2.77 (1.91 to 4.02) *	1.42 (0.90 to 2.25)
Frothy urine	13.51 (2.78 to 65.56) *	4.80 (0.82 to 28.17)
Halitosis	2.13 (1.54 to 2.95) *	1.40 (0.90 to 2.25)
Dry mouth	2.99 (1.92 to 4.64) *	2.36 (1.36 to 4.09) *
Mouth Ulcer	1.74 (1.20 to 2.51) *	1.04 (0.65 to 1.66)
Acne	0.88 (0.57 to 1.37)	-
Constipation	1.16 (0.82 to 1.63)	-
Cough	1.01 (0.65 to 1.55)	-
Dry throat	3.51 (2.27 to 5.41) *	2.20 (1.27 to 3.81) *
Sorethroat	1.27 (0.91 to 1.77)	-

* Statistical significance, p < 0.05

** No children with irritability also reported this symptom

Remedies used by parents for improving each of the aforementioned symptoms of "Hot Qi" were summarized in Table 4. The top five remedies were the increased consumption of water (86.8%), fruit (72.5%), soup (70.5%), the use of herbal beverages "five-flower-tea" (57.6%) and "selfheal fruit spike" (42.3%). Other commonly used remedies include the herbal beverage "Milk-Appetite Stimulant".

**Table 4:** Age differences in children given specific remedy

		Median age of Children (IQR)		P*
			
	N (%)	Given	Not Given	
Water	784(86.8)	7.0 (2.0 to 15.0)	7.0 (3.0 to 12.0)	0.953
Fruit	656 (72.5)	8.0 (4.0 to 14.0)	3.0 (0.83 to 10.0)	<0.001
Chinese Soup	637 (70.5)	8.0 (4.0 to 14.0)	3.0 (0.88 to 10.0)	<0.001
Five-flower-tea	521 (57.6)	10.0 (5.0 to 15.0)	3.9 (1.0 to 8.5)	<0.001
Selfheal Fruit Spike	382 (42.3)	10.0 (6.0 to 16.0)	4.5 (1.5 to 10.0)	<0.001
Western Medical Doctor	321 (35.5)	7.0 (3.0 to 14.0)	7.0 (3.0 to 12.0)	0.489
Appetite Stimulant	252 (27.9)	3.5 (1.5 to 6.9)	9.0 (4.5 to 15.0)	<0.001
Abrus Herb	231 (25.6)	10.0 (5.8 to 14.0)	6.0 (2.0 to 11.0)	<0.001
Millet Water	211 (23.3)	4.3 (2.0 to 11.0)	8.0 (3.5 to 13.0)	<0.001
Turtle Jelly	200 (22.1)	12.0 (8.0 to 17.0)	6.0 (2.0 to 11.0)	<0.001
Mulberry Leaf & Chrysanthemum Flower Tea	198 (21.9)	11.0 (7.0 to 16.0)	6.0 (2.0 to 11.0)	<0.001
Chinese Medical Doctor	182 (20.2)	11.0 (6.0 to 16.0)	6.0 (2.0 to 11.0)	<0.001
Mix Milk with Rice Water	142 (15.7)	3.0 (1.0 to 6.0)	8.0 (4.0 to 14.0)	<0.001
Bo Ying Compound	114 (12.6)	3.5 (1.75 to 6.0)	8.0 (3.33 to 14.0)	<0.001
24-Taste-Herb Tea	111 (12.4)	14.0 (9.0 to 18.0)	6.0 (2.67 to 11.0)	<0.001
Warm Salt Water	87 (9.6)	11.0 (7.0 to 14.0)	6.5 (3.0 to 12.0)	<0.001
Instant Chrysanthemum Tea	75 (8.3)	12.0 (8.0 to 18.0)	6.5 (3.0 to 12.0)	<0.001
Yin Chiao Chieh Tu Pien	47 (5.2)	16.0 (10.0 to 20.0)	6.5 (3.0 to 12.0)	<0.001

* Comparing the age of children "given" and "Not given" a specific remedy by Mann-Whitney test

The types of remedies given by parents were affected by the ages of their children (Table 4). Children given herbal beverages such as "five-flower-tea" and "selfheal fruit spike", abrus herbs, turtle jelly, mulberry leaf / chrysanthemum tea, instant chrysanthemum tea, and "24-taste-herb-tea", consumption of fruit, and soup were significantly older than those not given. In the contrary, children given remedies such as "Milk-Appetite stimulant", mix milk with rice water, and "Bo yin compound" were significantly younger.

## Discussion

"Hot Qi" was commonly used by parents in Hong Kong to describe symptoms of their children. The current study shed light on the meanings of the term "Hot Qi" as used by the lay public in a Chinese society and the treatment employed for alleviation of "Hot Qi".

Parents who were born in Hong Kong were more likely to use the term "Hot Qi" than those born in China and other part of the world. The association between "Hot Qi" and place of birth of the interviewed parent could be related to the intense advertisement of herbal beverages in the Hong Kong society. Herbal beverage companies promoted the concept of "Hot Qi" and advocated the effectiveness of herbal remedies in controlling "Hot Qi". The readily available Chinese herbal beverages in the market might have led to the widespread use of the term "Hot Qi". The other reason might be related to the fact that majority of adults in Hong Kong had first-hand experience with TCM [4,5] and most believed TCM to be an effective treatment. [6] The parental experience in TCM may prompt them to use the term "Hot Qi" to describe symptoms in their children.

The age of the children was another parameter useful for predicting whether parents used the term "Hot Qi". Parents seem more inclined to use the term "Hot Qi" to describe older children, median age of 7.0-year vs. median age of 1.0-year. This could be because parents became more confident with their childcare experience as the children get older and would let their children consume herbal products at an older age.

The most frequently mentioned symptoms of "Hot Qi" were eye discharge, sore throat, halitosis, constipation, and irritable mood. As allergic rhinitis with or without allergic rhinosinusitis is a common cause for nasal obstruction [7] / mouth breathing [8] / halitosis [9] whilst allergic conjunctivitis is often associated with eye discharge, [10] we suspected a major reason for "Hot Qi" is related to the underlying allergic rhinoconjunctivitis.

For the fifth most frequently mentioned symptoms of "Hot Qi" – irritability – we looked at the associated symptoms. Irritability and its associated symptoms (dry eyes, nasal obstruction, poor appetite, dry mouth, and dry throat) suggested possible upper respiratory system infection or allergic rhinitis. Dry mucosa complaints may well be related to the anti-cholinergic drugs prescribed to control the nasal symptoms of upper respiratory tract or allergic rhinitis. An increased consumption of liquid and fruits may help relieve some of the symptoms related to dry mucosa, but may not be appropriate for curing the underlying diseases.

Water, fruits, soups, and "five-flower-tea" and "selfheal fruit spike" were the top five remedies that parents used to alleviate the aforementioned symptoms of "Hot Qi". We found that these self-remedies used were generally appropriate for treating the symptoms (vide infra). Halitosis is caused by mouth breathing or poor dental hygiene or sinusitis. An increase in water consumption may help improve the dry mouth associated with mouth breathing and hence decrease the halitosis. Sore throat is usually caused by viral or bacterial infection, or by dry throat resulted from mouth breathing. Increased water and fruits consumption give the pharynx more fluid exposure to lessen the discomfort caused by sore throat. Constipation is associated with inadequate fluid intake and fibre intake. A greater intake of water and fruits would be effective to control constipation.

The Chinese herbal teas "five-flower-tea" and "selfheal fruit spike" were frequently used by parents for treating "Hot Qi". In TCM, both herbs are useful for clearing heat of the body and restoring the smooth flow of "Qi". Despite their popularity, we found no study that evaluated the beneficial effect of "self-heal fruit-spike" and "five-flower-tea" in human. In animal model study, the triterpenes extracted from selfheal fruit-spike (*Prunella vulgaris*) could inhibit the production of nitric oxide from cultured murine macrophages, suggested anti-allergic and anti-inflammatory effects. [11] In-vitro study on the triperpene alcohol extracted from *Chrysanthemum morifolium *(a component of "five-flower-tea") was reported to have anti-inflammatory effects. [12]The aqueous extract of *Lonicera japonica *demonstrated potent suppression of nitric oxide and tumor necrosis factor-alpha production in an activated macrophage-like cell line. [13] The extract of *Bombax malabaricum *was reported to have analgesic, antioxidant [14] and anti-*Helicobacter pylori *effects. [15] Oxymatrine, a alkaloids extracted from *Sophora Japonica *had anti-inflammatory effect, anti-virus effect as well as protecting hepatocytes and anti-hepatic fibrosis in murine. [16] Compound extracted from *Plumeria rubra *had molluscicidal, cytotoxic and antibacterial activity. [17] As most of the above herbs contain chemicals with anti-inflammatory, anti-allergy, anti-virus and anti-bacteria activity, those treatments for "Hot Qi" may actually treat the underlying atopic diseases or infection in children.

The main limitation of the current study was the potential sampling bias as the study was undertaken in only one public hospital and one private clinic. This was reflected by the fact that the percentage of low household monthly income between US$1282–3846 was significantly higher in the studied population compared with the general population. [18] (56.6% vs. 49.9%, Chi-squared test, p < 0.001) although the percentage of family on social security was similar to the general population. Hence, a territory-wide survey would give a more representative picture.

## Conclusion

"Hot Qi" is often used by Chinese parents to describe symptoms in their children in Hong Kong. Hong Kong-born parents and older children are main factors for parents to apply the term "Hot Qi" to describe symptoms of their children. The common symptoms of "Hot Qi" suggest infections or allergy.

## List of abbreviations

TCM = Traditional Chinese Medicine

CI = Confidence intervals

IQR = Inter-quartile range

## Competing interests

The author(s) declare that they have no competing interests.

## Authors' contributions

FYP participated in interview and preparation of the manuscript; DKN participated in the conceptualization and design of the study and final approval of the manuscript to be published; CHC participated in statistical analysis of the data; WLY, DC, KLK and PYC participated in the design of the study.

## Supplementary Material

Additional File 1 Brief description of the content: Questionnaire used in this study Click here for file
