# Supplementary material for: Parental use of the term "Hot Qi" to describe symptoms in their children in Hong Kong: a cross sectional survey "Hot Qi" in children
Source: J Ethnobiol Ethnomed. 2006 Jan 5;2:2. doi: 10.1186/1746-4269-2-2 (PMC1363719; doi:10.1186/1746-4269-2-2)
Supplement: Additional File 1 — Brief description of the content: Questionnaire used in this study [file 1746-4269-2-2-S1.doc]

**Survey on the Meaning of the term “Hot Qi” When Used by Chinese Parents**

# By Paediatric Department of Kwong Wah Hospital, Hong Kong

Age: □ <30 □ 30-40 □ 40-50 □ 50+

Sex: □ M □ F

Education Level: □ Primary □ Secondary □ College/University

Place of Birth: □ China □ Hong Kong □ Others

Monthly Household Income (HK dollars): □ CSSA □ <10K □ 10K – 20K □ 20K – 30K □ 30K– 40K □ 40K – 60K □ >60K

Age of Child: □ M □ F

□ Does the above mentioned child suffer from G6PD Deficiency?

**Have you ever employed the term “Hot Qi” to describe your child?**

-**□ No (Questionnaire finished. Thank you very much for your participation.)**

**-□ Yes.** **Please answer the following questions :**

Who gave the diagnosis? □ Yourself □ Chinese Medical Doctor □ Family & Friends □ Child Self-Report

Which type of “Hot Qi”? □ *Xu* (Deficiency) □ *Shi* (Excess) □ Don’t Know

Symptoms of “Hot Qi” (Please check all that apply):

□ Dry Eyes □ Poor Appetite □ Halitosis □ Dry Throat

□ Eye discharge □ Irritable □ Dry Mouth □ Sore Throat

□ Epitasis □ Yellowish Face □ Mouth Ulcers □ Others :___________

□ Nasal Obstruction □ Yellowish Sclera □ Acne

□ Running Nose □ Concentrated Urine □ Constipation

□ Gum Swelling □ Frothy Urine □ Coughing

When your children have “Hot Qi”, which of the listed treatment(s) would you use to improve the aforementioned symptoms? Please check (√) the box if you have tried the method of treatment before and use the visual analogue scale to rate its effectiveness :

If you believe that the treatment can effectively improve symptoms of “Hot Qi”, please mark “Χ” towards the right side of the line.

Example: No Effect I X I Very Effective

|  | Used | Effectiveness Rating |
| --- | --- | --- |
| Western Medical Doctor | □ | No Effect Very Effective mm |
| Chinese Medical Doctor | □ | No Effect Very Effective mm |
| Fruits | □ | No Effect Very Effective mm |
| Water | □ | No Effect Very Effective mm |
| Warm Salt Water | □ | No Effect Very Effective mm |
| Mix Milk with Rice Water | □ | No Effect Very Effective mm |
| Chinese Soup | □ | No Effect Very Effective mm |
| Chinese Medicine :  Appetite Stimulant (開奶茶)  Millet Water (薏米水)  5 Flower Tea (五花茶)  24 Tastes Herb Tea (廿四味)  Selfheal Fruit-Spike (夏枯草)  Abrus Herb (雞骨草)  .Turtle Jelly (龜苓膏)  Instant Chrysanthemum Tea  (菊花晶)  Mulberry Leaf & Chrysanthemum Tea (夏桑菊)  Yin Chiao Chieh Tu Pien  (銀翹解毒片)  Po Ying Pill (保嬰丹)  Others. Please Specified:  ___________ | □  □  □  □  □  □  □  □  □  □  □  □ | No Effect Very Effective mm    No Effect Very Effective mm  No Effect Very Effective mm  No Effect Very Effective mm  No Effect Very Effective mm  No Effect Very Effective mm  No Effect Very Effective mm  No Effect Very Effective mm  No Effect Very Effective mm  No Effect Very Effective mm  No Effect Very Effective mm  No Effect Very Effective mm |
